# An Evaluation of Patient Privacy Protection with Fuzzy Conjoint Analysis—A Case Study from Nurses’ Perspective

**DOI:** 10.3390/healthcare12131363

**Published:** 2024-07-08

**Authors:** Güney Gürsel, Nükhet Bayer, Ömer Turunç, Abdullah Çalışkan, İrfan Akkoç, Ayhan Demirci, Melike Çetin, Özlem Köroğlu

**Affiliations:** 1 Software Engineering Department, Faculty of Engineering and Architecture, Konya Food and Agriculture University, Konya 42080, Turkey; 2 Department of Health Sciences, Lokman Hekim University, Ankara 06530, Turkey; nukhet.bayer@lokmanhekim.edu.tr; 3 Business Administration Department, Faculty of Economic, Administrative and Social Sciences, Antalya Bilim University, Antalya 07110, Turkey; omer.turunc@antalya.edu.tr; 4 Health Management Department, School of Health Sciences, Toros University, Mersin 33140, Turkey; abdullah.caliskan@toros.edu.tr (A.Ç.); ozlem.koroglu@toros.edu.tr (Ö.K.); 5 Rectorate, Izmir Tinaztepe University, İzmir 35400, Turkey; dr.irfanakkoc@gmail.com; 6 Department of International Trade and Logistics, Faculty of Economic, Administrative and Social Sciences, Toros University, Mersin 33140, Turkey; ayhan.demirci@toros.edu.tr; 7 Department of Economics, Faculty of Economic, Administrative and Social Sciences, Antalya Bilim University, Antalya 07110, Turkey; melike.cetin@antalya.edu.tr

**Keywords:** evaluation, patient privacy, fuzzy conjoint analysis, similarity

## Abstract

Background: With the rapid improvement in healthcare technologies, the security and privacy of the most sensitive data are at risk. Patient privacy has many components, even when data are in electronic format. Although patient privacy has extensively been discussed in the literature, there is no study that has presented all components of patient privacy. Methods: This study presents a complete assessment framework, develops an inventory as an assessment tool, and examines the reliability and validity of the inventory. The study was carried out in three phases: conceptual framework development, inventory development, and an evaluation case study. Fuzzy conjoint analysis was used in the evaluation to deal with subjectivity and ambiguity. As a result of the evaluation, the case study institution was given a patient privacy maturity level between 1 and 5, where 1 is the worst and 5 is the best. Results: The case study evaluated the largest hospital in Turkey, which employs 800 nurses. Half of the nurses, 400, participated in the study. According to the literature, healthcare institutions do not invest enough in protecting patients’ privacy, and the results of the study support this finding. The institution’s maturity level was 2, which is poor. Conclusions: This study measured privacy maturity with many assessment components. The result of the assessment explains to patients and the public whether their data are secure or not. With the implementation of this maturity level, patients have an idea about which institution to choose, and the public can infer the reliability of institutions in terms of patient privacy.

## 1. Introduction

Patient privacy and security has become very popular, especially as electronic data have become a part of everyday healthcare. Although privacy and security are two different things, the term is used as a concept. Security is about how to protect, while privacy is about what to protect. Security is related to the storage and transmission of patient data with integrity, validity, and authenticity, while privacy is related to authorized access to and the disclosure of patient data [1,2].

Patient privacy can be defined as the right of individuals to hide/disclose their health-related data from and to anyone, and control the data regardless of who has them, while disclosing them for healthcare, treatment, or other reasons [3,4].

Patient health data are the most sensitive data of a person. If exposed, they can cause people to feel shame, embarrassment, insult, and discouragement, leading to losses of jobs and family. On the other hand, these data can be used for research, education, public health, insurance payments, etc., to improve healthcare services.

The health sector is placed in the top three in the yearly reported data security events [5]. Healthcare institutions are a direct threat to patient privacy, although they help patients by giving providing services, because of [5]:

- information system dependence,
- medical device connections,
- multiple software usage,
- multi-user shared devices.

Another internal threat is that medical staff and other employees have access to electronically stored or written patient records. Many staff fail to take even the simplest precautions due to negligence. User negligence is the leading cause of data security breaches [6,7].

Patient privacy can be violated due to the physical problems of health institutions, a lack of attention from healthcare professionals, or can be sacrificed to supposedly protecting patient against risks [8,9,10].

Patient data are transferred electronically for many purposes, such as the internet of things, homecare solutions, insurance, and public health purposes, etc., among systems, devices, and organizations. The fast improvement of medical technology also brings about new patient data sources such as biometric sensors [11]. Privacy leakage from medical wearable devices is another threat to patient privacy [12,13]. Haris, Haddadi, and Hui [14], examined the privacy leakage risks posed by mobile computing on mobile and wearable devices.

In exchanging patient data electronically with other systems, patient privacy is a major issue that has to be considered in health information exchange (HIE) [15]. Murdoch gave examples about the identification of patients from anonymized data in which protected health information is removed [16]. The study showed that protected data are also at stake, no matter whether an institution employs any policy or not.

Despite so many threats and risks to patient privacy, the majority of healthcare institutions do not invest enough in protecting patient privacy [16,17].

The protection of privacy and the collection, storage, and use of health data is a delicate balance. Therefore, the collection, storage, access, use, and disclosure of these data must be subject to regulations such as laws, institutional policies, technical infrastructure, etc.

Two big and important regulations on this issue are General Data Protection Regulation (GDPR) and Health Information Portability and Accountability Act (HIPAA).

GDPR imposes obligations onto organizations and people who want to target or collect data related to people in the EU [18,19]. Although it is not dedicated to health data, it is the toughest privacy and security law in the world.

The Health Information Portability and Accountability Act (HIPAA) is a dedicated and detailed patient privacy rule.

Protecting and safeguarding patient privacy is an ethical obligation of healthcare professionals and institutions, in accordance with ethical codes and principles [20]. But it must also be a legal obligation imposed by law, such as HIPAA and GPDR. This is because breaches can be intentional, unintentional, accidental, or negligent by healthcare professionals.

If there is no defined policy/principle/standard/requirement, it is not possible for healthcare institutions to protect patient privacy. There are many patient privacy scales/inventories in the literature for a variety of evaluation purposes, but each evaluates only one aspect, for example, staff-related characteristics, patient-related characteristics, system-related characteristics, etc. This deficiency prevents us from developing complete mechanisms including all aspects of patient privacy protection, because we cannot measure and determine the weak points. We cannot manage something that we cannot measure, as the management guru Peter Drucker famously said [21]. In addition, regulations fall behind the rapidly improving technologies they try to govern [16] by being inapplicable or impotent in terms of novel cases and technologies [4].

Because of these problems, the aim of this study is to introduce a complete framework for assessing the maturity of patient privacy protection in all aspects for healthcare institutions. The main objective is to evaluate the extent that a healthcare institution protects patient privacy. As the result of the evaluation, a maturity score is given to the institution under evaluation.

In this framework, the first step is to identify and document the arguments for protecting patients’ private data. In the next step, an inventory will be developed with the aim of measuring the level of maturity of healthcare institutions in the area of patient privacy protection. The sub-objectives of the study are listed below.

- A suggestion of the electronically stored minimum and maximum data sets needing to be protected.
- The access mechanisms to these data sets.
- The authorization mechanisms of healthcare staff.
- The education/awareness/informing mechanisms of healthcare staff about patient privacy.

These sub-objectives are very important, because, in all studies available in the literature, there are no clear definitions or access suggestions for arranging and managing accessing private sensitive data.

A case study was conducted in which nurses evaluated the institution in which they worked, because nurses are the dominating healthcare staff in number and are directly front facing towards patients.

## 2. Materials and Methods

### 2.1. Ethical Considerations

This study was ethically approved by the Lokman Hekim University Non-Invasive Research Ethical Committee on 13 September 2021 with the 2021/11 document.

### 2.2. Study Design

**Figure 1:**
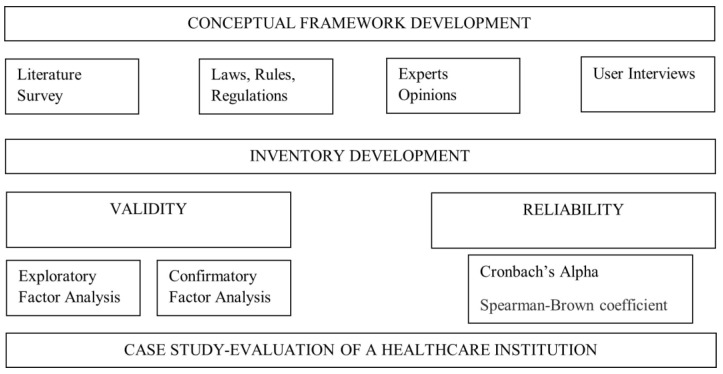
Study design.

The study is carried out in three steps. The first step is to establish the arguments for the protection of patients’ private data, i.e., to develop a conceptual framework; the second step is to develop an inventory of the level of maturity of the protection of patients’ private data and then to examine the validity and reliability of the inventory. In the last step, the scoring results of the inventory will be examined. The study design is shown in Figure 1.

### 2.3. Conceptual Framework

In the conceptual framework development, the literature, expert opinions, site interviews, white papers, laws and regulations, and many other related documents and views were examined, analyzed, and reviewed (Figure 1). As result, 32 variables under 5 dimensions were determined to be important for patient privacy. In this part, the details of the conceptual framework will be explained and examined.

The dimensions of the patient privacy protection maturity evaluation framework are:

- Management
- Users
- Patient
- Data
- Healthcare Information System (HCIS)

**Figure 2:**
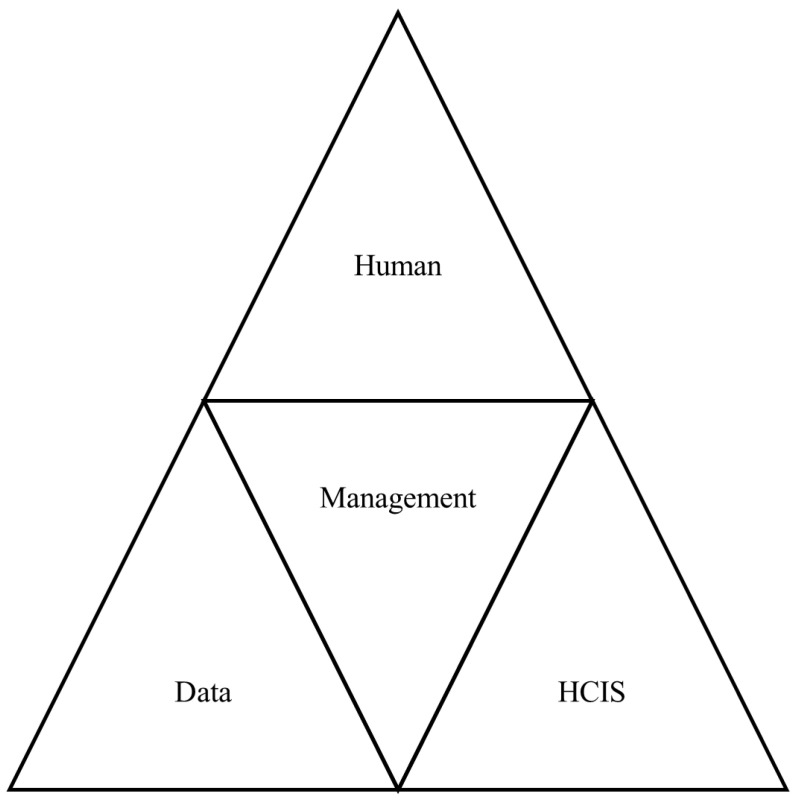
Privacy dimension hierarchy.

From the bottom up, the hierarchy of the dimensions used in the evaluation framework is given below in Figure 2. The dimensions are the key evaluation topics. Management is the top-most important part, so it is on the base. Without the support of management and the actions that need to be enacted by management, patient privacy and security cannot be guaranteed. Then comes the human, which is made up of users, patients, and managers. The third part is the data model. At the top, the policies and mechanisms in HCIS are important.

**Figure 3:**
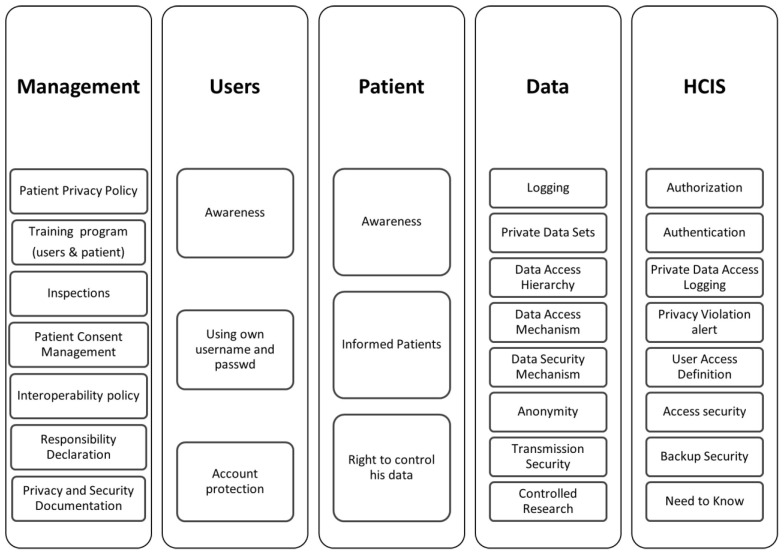
Privacy variables.

In Figure 3, under the dimensions (Figure 2), the variables of the evaluation framework are given. All the variables and mechanisms are presented in detail below.

Patient privacy policy: all healthcare institutions should have a written, approved, and publicly stated policy on patient privacy. The policy should clearly state the patient’s rights and the institution’s and staff’s responsibilities and penalties.

Training program (users and patients): All healthcare institutions should train their staff and patients in terms of privacy. In this training, staff should be trained about awareness, actions to undertake, actions not to take, possible threats, the possible outcomes of privacy violations, and possible penalties for both the faulty institution and faulty person. Patients should be trained in terms of awareness, their rights, complaint mechanisms, consent, and control over their private data.

Inspections: All healthcare institutions should conduct regular, scheduled, or immediate patient privacy inspections. There should be an effective carrot-and-stick mechanism at the end of each inspection. The results of these inspections should be made public.

Patient consent management: Unfortunately, “patient consent” is a problematic area. Patients are not mature enough to really decide on their consent. Either they are afraid of not receiving a good service and give consent although they do not want to, or they are afraid of something bad (in vain) and do not give consent. They should be informed properly about the procedures and usage of their data and the risks together with their realization rate, etc., to enable patients to give the right consent and not the wrong consent.

Interoperability policy: In the technology era, systems are not running alone. There are many other interacting systems and devices. For a HCIS to be interoperable with other systems and devices is a good virtue, but patient privacy and security should be considered and handled as a challenge. To accomplish this, all healthcare institutions should have a written, approved, and declared policy describing the way to interoperate with connecting devices and systems in data exchange in terms of patient privacy.

Responsibility declaration: All healthcare institutions should have a written, approved, and declared responsibility declaration, clearly stating its responsibilities and penalties in terms of patient privacy and security.

Privacy and security documentation: All healthcare institutions should have privacy and security documentation for guidance to ease the burden on humans (users/patients/managers). This document should contain standards, laws and regulations, and how-to descriptions.

Awareness: Awareness is a very important issue for the human factor of patient privacy and security. Without knowing exactly what an issue is and what it is not, one cannot take the necessary actions to address it. The human factor of healthcare institutions should be very well aware of patient privacy and security.

Use of own username and password: The most common violation of patient privacy and security is caused by users’ not using their own username and password in healthcare institutions. HCIS users are not sensitive about their usernames and passwords and give them to other users. As we have stated before, user negligence is the major data security breach source. Considering that all users have different authorizations, sharing usernames and passwords means that most users operate in the HCIS with authorization that they do not have.

Logging: All the daily operations performed by HCIS users should be stored in the HCIS database. This logging history has to be used for possible privacy and security violations, and can also be used for retrospective examination in the case of complaints or suspicions.

Private data sets: All healthcare institutions should have clearly defined, listed, and declared private data sets about patients. These private data sets should be subject to special mechanisms both in the HCIS and routine use. The proposed private data set derived from HIPAA can be:

- Names.
- All elements of dates (except year).
- Phone numbers.
- Fax numbers.
- E-mail addresses.
- Social security numbers.
- Medical record numbers.
- Health plan numbers.
- Account numbers.
- Certificate/license numbers.
- All means of vehicle numbers.
- All means of device identifiers.
- Web Universal Resource Locators (URLs).
- Internet Protocol (IP) addresses.
- All means of biometric identifiers.
- Any comparable images.
- Any other unique identifying numbers.

Data access hierarchy: Having clearly defined the private data set, all elements of the data set should have an access hierarchy level. This means that all elements should be indicated by means of access security importance levels. An example of such a data access hierarchy is given in Table 1.

User access definition: By user access definition, the healthcare institution defines the level of HCIS users with their user access definition. A data access hierarchy and user access definitions can help institutions to manage which user can access which private data set elements. An example is given Table 2.

It is seen that physicians can access the entire data set given in Table 1 (they have an access definition of 7, which is equal to or greater than all the data access hierarchy levels) when the system combines user access definitions and data access hierarchy. Technicians, lab technicians and office workers cannot access any private data set elements.

Data security mechanism: A data security mechanism is a collection of measures to protect data from unauthorized access and malicious attacks, such as the secure protection of hardware, biometric measures, firewalls, etc.

Anonymity: Anonymity is an important measure to protect patient privacy. In particular, research studies, data exchange, consultations, billing, etc., procedures are very sensitive applications and prone to privacy violations. To protect privacy, anonymity is a good in such applications.

Transmission security: When transferring patient data, the institution should be aware of two stages: the time the data are in transit, and the time the data are sent and received. There must be mechanisms in place to ensure that the data are not viewed or modified during the time they are in transit.

Controlled research: Research is very important for the development of new technologies/advances/prognoses, etc., but there is a danger of the abuse and misuse of patient private data. To prevent privacy violations and enable research, there must be a control mechanism, and the best solution could be anonymity.

Authorization and authentication: These issues are very well known and do not need further definition. Authorization should be enforced according to user access definition.

Private data access logging: Any access to private data sets should be exclusively logged, separately from the logging defined above. With this special logging, privacy violations can be spotted online and in a timely manner.

Privacy violation alert: In HICS, there can be smart, online alert mechanisms about potential patient privacy violations to enable management to prevent possible cases.

Backup security: While backing up the data in the HCIS database, there should also be mechanisms to prevent privacy violations and protect privacy.

Need to know: This is a very important principle that can be used to protect security. In access to private data sets, the construction of user access definitions can be referenced according to a need-to-know principle. If a user does not need to know an element of the private data set, then the access should not be given.

### 2.4. Data Collection

The study was conducted in the biggest hospital in Türkiye, with 800 nurses employed. It is a public hospital with a big number of inpatient and outpatient visitors. Volunteered nurses (400) participated in the study with face-to-face interviews, and 307 completely answered surveys were analyzed.

### 2.5. Statistical Analysis

Analysis and measurements were conducted by the means of the Statistical Package for the Social Sciences (SPSS) and Analysis of Moment Structures (AMOS).

#### 2.5.1. Content Validity

To measure the content validity of the inventory, experts in the field of nursing and the fields of measurement and evaluation were asked to evaluate each expression in terms of its comprehensibility and compatibility. The inventory was arranged and modified according to the feedback from the experts.

#### 2.5.2. Structure Validity

At first, an Exploratory Factor Analysis (EFA) was performed prior to a Confirmatory Factor Analysis. In the EFA, Kaiser–Meyer–Olkin (KMO) and Bartlett’s tests were used as prerequisites to determine the items’ fitness in the Confirmatory Factor Analysis (CFA); in addition, communalities were examined. Later, data were analyzed with the CFA to confirm validity, reliability, and goodness-of-fit testing. For structural validity, fits tests, Comparative Fit Index (CFI), Goodness of Fit Index (GFI), Adjustment Goodness of Fit Index (AGFI), and Normed Fit Index (NFI) were used.

In CFA, the most frequently used indicators are chi-square, root- mean-square error (RMSE), and root-mean-square error of approximation (RMSEA) [22]. Chi-square has proven to be sensitive to sample size, and tends to be more significant as the sample size grows. To eliminate this handicap and avoid bias, χ^2^/df should be used together with other goodness of fit indexes (AGFI, GFI, CFI, NFI, IFI, and RMSEA) [23]. The CFA fit indexes are given in Table 3 [23,24].

#### 2.5.3. Reliability

The reliability of the inventory was examined by Guttman’s, Cronbach’s Alpha, and Spearman–Brown coefficients, which were used to measure the internal consistencies of the results. Values greater than 0.70 are accepted as having a good reliability. Values closer to 1 are better.

#### 2.5.4. Patient Privacy Protection Maturity Inventory

The Patient Privacy Protection Maturity Inventory is the data collection tool of the patient privacy protection maturity evaluation framework, developed specifically for this study. In the inventory, 30 questions exist in which evaluators—in this study, healthcare staff—can express their evaluations/answers using a five-point Likert scale, Strongly Agree, Moderately Agree, Not Sure, Moderately Disagree, and Strongly Disagree.

#### 2.5.5. Evaluation

Data were captured using the Patient Privacy Protection Maturity Inventory. Fuzzy logic methodology, namely, Fuzzy Conjoint Analysis (FCA), was used for calculations of the inventory results. The Likert scale context, having ambiguity and multiplicity in meaning, is very suitable for fuzzy logic methodologies. FCA is the statistical and multi-criteria evaluation method used to determine the value of a product that one appraises and one’s preferences related to the criteria employed [25].

The literature has proposed using fuzzy logic and conjoint analysis together to model the subjective preferences of evaluators [26].

The Likert scales of the inventory were converted into fuzzy triangular numbers (fuzzification). In fuzzification, the crisp values and linguistic variables of the method are converted into fuzzy sets. In Table 4, the triangular numbers used to fuzzify the inventory ratings captured are given. Using Table 4, the Likert scales used in the inventory were converted into fuzzy triangular numbers to use in Formula (1). Each linguistic variable (Likert scale) is characterized by a triangular fuzzy number that has an approximate value ranging between 0 and 1 for importance and between –1 and 1 for the rating. Assuming that the distance between the linguistic variables is equal, the mean values for importance are determined as: not important = 0, not so important = 0.25, averagely important = 0.5, important = 0.75, and very important = 1.

The membership of each inventory variable to the defined linguistic variables (our Likert scales), µ*R*(*Xj*″, *F*″ *j*), is computed with;

(1) µRXj,Fj=∑i=1nwi∑wi·Xi

where:

- *wi* is the answer given by the *i*-th participant,
- *∑Wi* is the sum of the answers given to the *i*th inventory item,
- *wi*/∑*Wi* is the weight of the *i*-th participant,
- *Xi* is the corresponding fuzzy set of the *i*-th respondents (if the answer is “Moderately disagree”, then *Xi* is (0, 0.25, 0.5)),
- *Fj* is the *j*th inventory item,
- *n* is the total number of answers.

This membership gives us the fuzzy set of each inventory variable. It is time for a comparison of these sets with the defined fuzzy sets and to determine to which linguistic variable the given response is closer. Here, the main aim is to capture which original linguistic variable is the closest to the final fuzzy set obtained from the participants’ answers. Similarity is measured by:

(2) $$\mathrm{Sim}(Ri\left(yj,A\right),F\left(xj,l\right)=\frac{1}{\left[1+\sqrt{\sum_{j=1}^{n}(\mu Ri}\left(yj,A\right)-\mu F\left(xj,l\right){)}^{2}\right]}$$

where:

- *Ri* (*yj*, A) is the fuzzy set determined by 2 (Formula (2)),
- F (*xj*, l) is the standard fuzzy sets defined (Table 1)

In the last step of the evaluation, the patient privacy maturity score of the evaluated institution is determined. This determination is made according to the similarity values. If the similarity is close to the worst Likert scale, “Strongly Disagree”, then the patient privacy maturity score is maturity level 1, when close to “Moderately Disagree”, it is maturity level 2, when close to “Not Sure”, it is maturity level 3, when close to “Moderately agree”, it is maturity level 4, and when close to “Strongly Agree”, it is maturity level 5. In terms of patient privacy, the higher the maturity score of the healthcare institution, the more mature it is.

## 3. Results

In total, 400 volunteered nurses participated in the study, while 307 of them were (who fully completed the evaluation) included, aged between 22 and 61 with an average age of 35.79. A total of 19 participants were female (6.20%) and 288 male (93.80%), while 99 were married (32.20%) and 208 single (67.80%). In total, 39 participants expressed their education as high school graduate (12.70%), 36 as BS (11.70%), 203 as university (66.10%), 27 as MS (8.80%), and 2 as Ph.D (0.70%). A total of 29 participants were working in the emergency department (9.40%), 12 in operations (3.90%), 31 in surgical departments (10.10%), 137 in internal medicine (44.60%), 41 in administration (13.30%), and 57 in intensive care (18.60). It was seen that 13.30 of the users were from management.

Before the factor analysis, reliability was measured. Cronbach’s Alpha, our reliability coefficient, was measured for all 30 variables as 0.973, the Spearman–Brown coefficient was 0.949, and Guttman’s coefficient was 0.973. Cronbach’s Alpha, Spearman–Brown coefficient, and Guttman’s coefficient according to the dimensions are given in Table 5. These coefficients were used to measure the internal consistencies of the results, where greater than 0.70 is acceptable and closer to 1 is better.

As a pre-requisite to CFA, the KMO Measure of Sampling Adequacy (MSA) value, 0.959, was measured to see if the variables fit the factor analysis. The measured value was very close to 1. The Bartlett’s Test of sphericity proved to be significant (*p* = 0). These proved that the research variables were appropriate for a factor analysis [27].

In the EFA, the five dimensions explained a total of 72.175% of the variance among the items in the study. Varimax rotation analysis was evaluated seven times. All communalities were over the required value of 0.500 (between 0.504 and 0.822).

In the CFA, the fit values obtained are given in Table 6.

The similarity coefficients and the closest Likert scales of all inventory items are given in Table 7. It is seen that all the items were closer to ‘Moderately disagree’. The highest coefficients are marked as bold. In Table 6, the highest value is taken, in other words, the value closest to 1, as explained in the Section 2. The corresponding Likert scale of the value is the most similar choice we are looking for. Table 7 and Table 8 contain the main results of the study, which give the maturity score. The study results will be discussed using the results in these tables.

The similarity coefficients and closest Likert scales according to the dimensions are given in Table 8. As in the variables, all were close to the Likert scale ‘Moderately disagree’.

## 4. Discussion

In this study, a complete patient privacy protection maturity evaluation framework is introduced. In this framework, healthcare institutions are evaluated in five dimensions considering 30 different criteria that have direct impacts on patient privacy protection. This evaluation was executed by the means of a patient privacy maturity inventory, developed specifically for this study, to capture the evaluation data.

According to the reliability analysis, it can be said that the inventory is reliable for measuring the privacy protection maturity of a healthcare institution. All reliability coefficients (Guttman’s, Cronbach’s Alpha, and Spearman–Brown) were near to 1, showing a very high reliability.

Regarding the validation of the proposed framework, the EFA showed a high explanation ratio with 72.175%, together with high communality scores. CFA proved to be almost a perfect fit, except the AGFI value, which had an acceptable fit.

It seemed as if all participants agreed on the results. The similarity values were very close to 1, changing between 0.76 and 0.86 (in 27 variables, >0.8, in 9 variables, greater than 0.84), as if almost all users gave the same answer. This shows a complete agreement on the evaluation of the institution.

According to the evaluation results, the institution’s patient privacy protection maturity score appeared as “maturity level 2” in all items and dimensions. This means almost no privacy policy, no training program, no inspections, no documentation, no defined private data sets, and no data access mechanisms. This result is compatible with the motivation of the study. If there is no principle/standard/requisition/prerequisite defined, it is not possible for healthcare institutions to protect patient privacy. We have said that securing and ensuring patient privacy is an ethical obligation of healthcare professionals and institutions [21]. In addition, we have also said that such an obligation must be imposed by laws just like in HIPAA and GPDR. The results of this study support these claims. Ethics can be avoided, especially when human life, health, or some other well-intentioned excuse is on the agenda, and these excuses can provide cover for potential violations. As noted above, in the absence of legal compulsion, healthcare institutions do not invest enough in protecting patient privacy [28] or spend enough resources [1].

Most privacy violations are discovered by the individual (patient/staff/etc.) whose privacy is violated, not by an analyst or a smart mechanism [5]. That shows there can be many more violations that remain undetected because nobody notices.

This study shows that, to meet the patient privacy requirements of the evaluation, institutions should invest in humans and HCIS by spending time and money and constructing a body to plan and investigate. This study also shows that institutions do not take the required measures to detect and prevent patient privacy violations in time, before they happen; most violations of patient privacy are seen when a dedicated investigation is performed, and it is usually too late because the violation has already occurred, and harm is experienced [5].

To prevent this, the required measures must be taken, periodic investigations should be performed, and the whole system, with all components, should be evaluated.

Managing a healthcare institution with the perfect level of patient privacy protection maturity requires a complete evaluation to prove this. If it is not at the desired level, we must find out the missing or lacking points. This is possible only if one evaluates the scope of patient privacy. This inventory may give institutions the ability to measure where they are in terms of patient privacy protection.

The results of this study prove that the framework and inventory can be used as a measure for healthcare institutions, and it can also be used to construct policies and standards. This study is only a case study, but it can be applied to different healthcare institutions and verify the model’s accuracy with more cases. But with the current obligations and regulations, this study shows that the result would not be different.

For further research, the framework can be used to evaluate more healthcare institutions together and analyze the comparative results. The evaluators can be all healthcare staff instead of only nurses, and again, comparative results between staff can be analyzed. Another version can use professional evaluators. These evaluators can be trained before the study, then the same team can evaluate different types and sizes of healthcare institutions.

The proposed framework can give detailed information to the evaluator. While it can be evaluated for a whole HCIS, it can also be filtered according to user profiles. The dimensions can be filtered instead of performing the whole evaluation. Only the user or management could be evaluated. Additionally, a lack of communication can also be a result of such a study. If a variable is expected to give a high maturity, but the result is the opposite, then we must seek the answer in communication. This means that either users are not well aware of the virtue of the system or the information about that variable is faulty.

The inventory can be used as an evaluation tool by ministries of health. It can also be used by management to measure their maturity level periodically to see improvements or declines if available. Good results can be used as a level attraction for patients in the commercial or public relation activities of hospitals.

## 5. Conclusions

The most significant contribution of this study is that it provides a complete evaluation framework for the protection of patient privacy. As stated in the introduction, although privacy evaluation in healthcare is widely discussed and many evaluation and protection methods are introduced, evaluations are only conducted considering one or a few aspects. In fact, privacy is a more complicated issue and has many elements that need to work together and complement each other to protect patient privacy. These privacy-complementary elements are presented in this study as a further contribution to the scientific literature.

The framework takes a screenshot of the current situation in all aspects of patient privacy protection, unlike studies in the literature, which concentrate on one or a few aspects.

The framework evaluates healthcare institutions and determines their patient privacy maturity score. The possible scores are maturity levels from 1 to 5, in which a higher score is better. By the means of this concrete output, the level of maturity of any healthcare institution can be measured, points for improvement can be understood, and in the next evaluation, progress in the level of maturity can be seen.

The framework enables us to spot and determine the weak sides and gaps in the patient privacy protection of an institution. It can point out potential privacy violation issues. This evaluation can be performed periodically to examine the maturity improvement in the scope of patent privacy. In addition, we can compare different healthcare institutions in terms of patient privacy protection and rank them from the highest level to the lowest or vice versa. This comparative analysis can be particularly useful for chain healthcare groups, which have more healthcare facilities and need to make internal comparisons and set standards.

Including only nurses in the research constitutes a limitation of the study. In this context, including all employees of the hospital, including physicians, technicians, attendants, and other employees, and comparing the results with the results obtained from other employees may provide broad perspectives. This study becomes a nurses’ perspective in terms of patient privacy.

Another limitation or further study point can be the involvement of management, being the base dimension of the evaluation. Management can evaluate the institution using the framework. Management may also improve or arrange the dimensions or variables of the evaluation framework according to the focus points or priorities of the institution.

## Figures and Tables

**Table 1:** An example of data access hierarchy.

Data	Hierarchy Level
Name	7
Phone Number	6
Certificate number	4
Plate Number	4
Social Security Number	7

**Table 2:** An example of user access definition.

Data	Hierarchy Level
Physician	7
Nurse	5
Technician	2
Lab technician	2
Office Worker	1

**Table 3:** CFA fit indexes.

	Perfect Fit	Acceptable Fit
AGFI	0.90 ≤ AGFI ≤ 1.00	0.85 ≤ AGFI ≤ 0.90
GFI	0.95 ≤ GFI ≤ 1.00	0.90 ≤ GFI ≤ 0.95
CFI	0.95 ≤ CFI ≤ 1.00	0.90 ≤ CFI ≤ 0.95
NFI	0.95 ≤ NFI ≤ 1.00	0.90 ≤ NFI ≤ 0.95
RMSEA	0.00 ≤ RMSEA ≤ 0.05	0.05 ≤ RMSEA ≤ 0.08
χ2/df	2 ≤ χ^2^/df ≤ 3	3 ≤ χ^2^/df ≤ 5

**Table 4:** Triangular fuzzy numbers assigned to Likert scales (linguistic variables).

Data	Hierarchy Level
Strongly Agree	0.75, 1, 1
Moderately Agree	0.5, 0.75, 1
Not Sure	0.25, 0.5, 0.75
Moderately Disagree	0, 0.25, 0.5
Strongly Disagree	0, 0, 0.25

**Table 5:** Reliability coefficient values.

Dimension	Cronbach’s Alpha	Spearman-Brown	Guttman’s
Management	0.929	0.870	0.930
User	0.834	0.830	0.834
Patient	0.853	0.768	0.856
Data	0.930	0.906	0.930
Information system	0.925	0.903	0.926

**Table 6:** CFA fit indexes obtained in the study together with reference values.

	Perfect Fit	Acceptable Fit	Study Value
AGFI	0.90 ≤ AGFI ≤ 1.00	0.85 ≤ AGFI ≤ 0.90	0.876
GFI	0.95 ≤ GFI ≤ 1.00	0.90 ≤ GFI ≤ 0.95	0.954
CFI	0.95 ≤ CFI ≤ 1.00	0.90 ≤ CFI ≤ 0.95	0.969
NFI	0.95 ≤ NFI ≤ 1.00	0.90 ≤ NFI ≤ 0.95	0.963
RMSEA	0.00 ≤ RMSEA ≤ 0.05	0.05 ≤ RMSEA ≤ 0.08	0.497
χ^2^/df	2 ≤ χ^2^/df ≤ 3	3 ≤ χ^2^/df ≤ 5	2.87

**Table 7:** Similarity values of the inventory variables.

Variable	Similarity	Strongly Disagree	Moderately Disagree	Not Sure	Moderately Agree	Strongly Agree
M1	Moderately Disagree	0.718218	**0.784835**	0.519829	0.426298	0.384119
M2	Moderately Disagree	0.699176	**0.80075**	0.532724	0.43434	0.390028
M3	Moderately Disagree	0.650733	**0.843053**	0.564349	0.449726	0.398236
M4	Moderately Disagree	0.709998	**0.788945**	0.52799	0.430863	0.386971
M5	Moderately Disagree	0.684141	**0.818907**	0.540279	0.437499	0.39033
M6	Moderately Disagree	0.628925	**0.828878**	0.587218	0.461348	0.403716
M7	Moderately Disagree	0.668136	**0.844412**	0.546092	0.439264	0.390171
M8	Moderately Disagree	0.66005	**0.842132**	0.556088	0.444531	0.393638
M9	Moderately Disagree	0.668635	**0.827864**	0.552673	0.443886	0.393705
U1	Moderately Disagree	0.733799	**0.766653**	0.514777	0.424011	0.382616
U2	Moderately Disagree	0.650898	**0.837291**	0.565584	0.451363	0.39905
U3	Moderately Disagree	0.642923	**0.8332**	0.572724	0.456872	0.403466
U4	Moderately Disagree	0.668369	**0.834325**	0.550549	0.442125	0.393091
P1	Moderately Disagree	0.663697	**0.827674**	0.557226	0.446755	0.396578
P2	Moderately Disagree	0.691294	**0.807406**	0.538052	0.436991	0.391008
P3	Moderately Disagree	0.642528	**0.843339**	0.571963	0.452661	0.398293
P4	Moderately Disagree	0.624436	**0.823395**	0.590312	0.466845	0.409217
P5	Moderately Disagree	0.698044	**0.801263**	0.533885	0.434788	0.388898
D1	Moderately Disagree	0.6586	**0.840305**	0.558345	0.445075	0.393691
D2	Moderately Disagree	0.62853	**0.838854**	0.585223	0.459092	0.402227
D3	Moderately Disagree	0.647867	**0.861636**	0.561251	0.446213	0.393621
D4	Moderately Disagree	0.630309	**0.831065**	0.585698	0.45986	0.403015
D5	Moderately Disagree	0.650272	**0.857155**	0.560497	0.445853	0.393649
D6	Moderately Disagree	0.648802	**0.847849**	0.564973	0.448839	0.396096
HCIS1	Moderately Disagree	0.627952	**0.860448**	0.579846	0.455302	0.39888
HCIS2	Moderately Disagree	0.671541	**0.832493**	0.547898	0.440915	0.39221
HCIS3	Moderately Disagree	0.663280	**0.839029**	0.553546	0.444322	0.394377
HCIS4	Moderately Disagree	0.624389	**0.836324**	0.589115	0.461753	0.404283
HCIS5	Moderately Disagree	0.637784	**0.851736**	0.573845	0.452763	0.397918
HCIS6	Moderately Disagree	0.654471	**0.847441**	0.559831	0.445748	0.394069

**Table 8:** Similarity values of the framework dimensions.

Dimension	Similarity	Strongly Disagree	Moderately Disagree	Not Sure	Moderately Agree	Strongly Agree
Management	Moderately Disagree	0.676446	**0.819975**	0.547471	0.440862	0.392324
User	Moderately Disagree	0.673997	**0.817867**	0.550909	0.443593	0.394556
Patient	Moderately Disagree	0.664	**0.820615**	0.558287	0.447608	0.396799
Data	Moderately Disagree	0.644063	**0.846144**	0.569331	0.450822	0.39705
Information System	Moderately Disagree	0.64657	**0.844579**	0.567347	0.450134	0.396956

## Data Availability

The raw data supporting the conclusions of this article will be made available by the authors on request.
